# Development and Characterization of Plant‐derived Aristatoside C and Davisianoside B Saponin‐loaded Phytosomes with Suppressed Hemolytic Activity

**DOI:** 10.1002/open.202300254

**Published:** 2024-03-11

**Authors:** Sebnem Ercelen, Bunyamin Bulkurcuoglu, Mustafa Oksuz, Ayse Nalbantsoy, Nazli Boke Sarikahya

**Affiliations:** ^1^ Life Sciences Bionanotechnology Lab. Scientific And Technological Research Council of Türkiye (TUBITAK) Marmara Research Center (MRC) Gebze 41470 Kocaeli/ Türkiye; ^2^ Hamidiye Faculty of Medicine Department of Biophysics University of Health Sciences Üsküdar 34668 İstanbul/ Türkiye; ^3^ Institute of Biotechnology Gebze Technical University Gebze 41400 Kocaeli/ Türkiye; ^4^ Faculty of Pharmacy Biochemistry Department Mersin University Yenişehir 33160 Mersin/ Türkiye; ^5^ Faculty of Engineering Department of Bioengineering Ege University Bornova 35040 İzmir/ Türkiye; ^6^ Faculty of Science Department of Chemistry Ege University Bornova 35040 İzmir/ Türkiye

**Keywords:** cancer, drug delivery, hemolytic activity, phytosome formulation, saponins

## Abstract

Saponins are glycosides widely distributed in the plant kingdom and have many pharmacological activities. However, their tendency to bind to cell membranes can cause cell rupture, limiting their clinical use. In the previous study, aristatoside C and davisianoside B were isolated from *Cephalaria* species. Cytotoxicity assays showed that they are more active on A‐549 cell lines than doxorubicin but caused hemolysis. In the current research, aristatoside C and davisianoside B were loaded to phytosomes called ALPs and DLPs respectively, and characterized for particle size, zeta potential, encapsulation efficiency, release kinetic, hemolytic activity, and cytotoxicity on A‐549 cell line. DLPs maintained the cytotoxic activity of the free saponins against A‐549 cells with IC50 of 9,64±0,02 μg/ml but dramatically reduced their hemolytic activity. Furthermore, temperature and time‐dependent stability studies based on the size and zeta potential of ALPs and DLPs revealed that the phytosomes have sustained release properties over 2 weeks. Overall, DLPs displayed cytotoxicity against A‐549 cells with minimal hemolysis and sustained release, highlighting their potential as nanotherapeutics for clinical applications.

## Graphical abstract

Phytosomal formulations of aristatoside C and davisianoside B compounds isolated from *Cephalaria Aristata* and *Cephalaria davisina* plants were prepared using the thin film hydration method. Physical characterization studies of the prepared phytosomal saponin formulation were carried out. The cytotoxic activity of phytosomal formulations was examined. The suppression of hemolytic activity of phytosomal formulations on erythrocytes caused by free saponins was investigated.

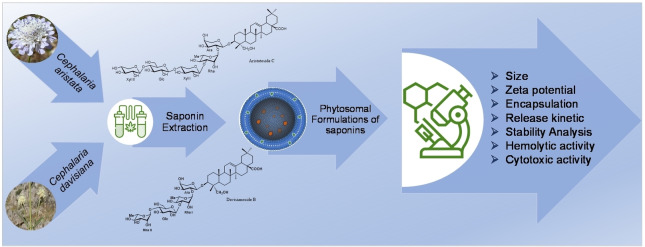

## Introduction

Plant‐derived saponins are naturally occurring glycosidic compounds characterized by either a steroidal or a triterpenoid aglycone and one or more monosaccharide units.^[1]^ Saponins have strong surface‐active properties due to the presence of polar (sugar) and non‐polar (steroid or triterpene) groups. These structural features are responsible for many negative and beneficial biological effects of saponins, such as hemolytic and anticancer activities.^[2]^ Triterpenoid glycosides a type of saponin, have diverse pharmacological properties and are used traditionally as well as industrially.^[1]^ These molecules display a wide range of biological activities such as cytotoxicity,^[3]^ antitumor,^[4]^ antiviral,^[5]^ hemolytic,^[6]^ and immunomodulatory activities.[^7^, ^8^] The cytotoxicity and anticancer role of saponins have been evaluated in many studies. The cytotoxic effect of most saponins is attributed to their ability to induce the apoptotic process in tumor cells, usually via the intrinsic pathway. Additionally, non‐apoptotic processes like cell cycle arrest, stimulation of autophagic cell death, inhibition of metastasis and cytoskeleton disruption are also involved in the cytotoxic activity of saponins.^[3]^ Liu et al. explored the inhibition properties of *Conyza blinii* saponins (CBS) isolated from the medicinal plant *Conyza blinii H.L'ev*. of autophagy, a promising therapeutic strategy for cancer treatment.^[9]^ It has been reported that CBS is an effective inhibitor of autophagy in HeLa cells and that its anti‐cancer activity is likely to be due to this property. Lu et al. investigated the anti‐tumor effect of liposomes loaded with thymosaponin A (TAIII), a steroid saponin found in Anemarrhena asphodeloides, and modified with an anti‐CD44 antibody.^[10]^ TAIII has potential as an anti‐tumor drug candidate, however, it's *in vivo* antitumor activity is limited by its hydrophobicity and low bioavailability. *In vitro* and *in vivo* biodistribution and ex vivo imaging studies in HepG2 tumor‐bearing mice have shown that a liposomal formulation of TAIII increases the plasma half‐life, reduces the systemic clearance, and improves bioavailability. Sarıkahya et al. investigated the cytotoxic, hemolytic, and immunomodulatory activities of nineteen triterpenoid saponins obtained from the aerial parts of eight Cephalaria species from Türkiye. HeLa, A‐549 and HEK293 cells were used for the cytotoxic assays of the saponins. Aristatoside C and davisianoside B displayed significant inhibitory effects on A‐549 and HeLa cells, while almost all saponins, including aristatoside‐C and davisianoside B, caused significant hemolysis on human erythrocytes. The reason for the hemolytic activity of saponins is thought to be the affinity of the aglycone part to membrane sterols, especially to cholesterol with which they form insoluble complexes.^[11]^ Many studies have shown that saponins may cause hemolysis by forming pores in cell membranes.[^12^, ^13^] It was hypothesised that saponins interact with the lipid membranes of cells and form insoluble complexes with cholesterol. This leads to the disruption the cell membrane, formation of pores, increase cell permeability and subsequent loss of hemoglobin in the extracellular environment.[^14^, ^15^] It was reported that changes in membrane proteins were also observed during erythrocyte permeabilization with saponins.^[16]^ The alterations in the erythrocyte membrane led to the formation of pores and hemolysis, most likely via a colloid‐osmotic mechanism.^[17]^

Besides their various superior preventive and therapeutic properties, clinical usage of saponins is only feasible if their hemolytic features are eliminated. Suppression of hemolytic activity of saponins could be achieved if saponins are loaded into a drug carrier by maintaining or increasing their biological activities for an extended period.^[12]^ According to recent studies, nanovesicles as drug carrier systems have become very attractive for drug delivery due to their enhanced biocompatibility, prolonged circulation time, and cost‐effectiveness. Phytosomes are nanovesicular structures with high stability due to chemical links between phospholipid molecules and phytoactive agents.[^18^, ^19^] The phytosome‐based drug delivery approach involves converting polyphenolic phytoconstituents, which are both challenging to dissolve and highly toxic, into a vesicular dosage form by interacting with phospholipids in an aqueous environment. These formulations have improved pharmacological and pharmacokinetic properties compared to commonly used preparations.^[20]^ The hemolytic activity of saponins can be minimized by encapsulating them into phytosomes and preserving their cytotoxic effects on cancer cells.^[21]^ Clearance of drugs from the body in a short time is a severe problem for the success of the therapy. The sustained release of therapeutics from phytosomes is an auspicious feature for their translation into clinics.^[21]^ Currently, several phytosome formulations are in clinical use.^[22]^ One of them is in phase 3, which is the quercetin‐loaded phytosome formulation as a dietary supplement to boost natural immunity to prevent COVID‐19 disease.^[23]^

In this study, we prepared phytosomal formulations of previously isolated and characterized saponins: aristatoside C and davisianoside B to reduce their hemolytic activity while retaining their cytotoxic activity. Physicochemical parameters such as size, zeta potential, and time‐dependent stability at +4 °C and +25 °C were investigated. The encapsulation efficiency and sustained release profile of both phytosomal formulations were investigated. The hemolytic activity of these formulations against human erythrocytes and their cytotoxic activity on A‐549 cells were evaluated.

## Results and Discussion

### Particle Size and Zeta Potential Analysis of ALPs and DLPs

Dynamic (DLS) and phase analysis (PALS) light scattering methods were carried out to evaluate particle size, polydispersity index (PDI) and zeta potential analysis for the ALPs and DLPs. ALPs and DLPs were prepared with sizes between 100–200 nm, the preferred range for biological applications (Table 1). The size distribution of EP, ALP and DLP is quite low and the approximate PDI value was found to be around 0,2. Phytosomal formulations of saponins have negative zeta potentials owing to their soy lecithin phospholipids.^[24]^ Overall, the data obtained for ALPs, DLPs, and EPs showed that the average size of all saponin formulations is about 115 nm with a fine size distribution. Zeta potential values were around −100 mV for all three phytosomes. Zeta potential values outside the −30,0 to +30,0 mV range demonstrate increased stability and long‐term storage features of nanocarriers.^[25]^

**Table 1:** Particle size, PDI, and zeta potential values of saponin‐loaded phytosomes.

	Particle Size (nm)	PDI^[a]^	Zeta Potential (mV)
ALPs	121,06±1,01	0,248±0,004	−106,8±0,4
DLPs	110,64±0,37	0,255±0,014	−88,1±2,8
EPs	119,64±0,90	0,211±0,01	−98,5±0,8

[a]: Polydispersity index.

### Encapsulation Efficiency (%EE)

The encapsulation efficiency of aristatoside‐C and davisianoside B loaded to phytosomes was calculated using equation (1) based on the spectrophotometric method (Table 2). This formula calculates the encapsulation efficiency by taking the total amount of saponin used to make the phytosomes, subtracting the number of free saponin molecules remaining in the sample, and dividing by the total amount. The encapsulation efficiencies for both saponins were very high, making them suitable for biological applications.

**Table 2:** Saponin encapsulation efficiencies of phytosomes.

	Encapsulation Efficiency (%)
ALPs^[a]^	68,0±5,2
DLPs^[b]^	79,5±3,1

[a] ALPs: aristatoside C loaded phytosomes. [b] DLPs: davisianoside B loaded phytosomes.

### Sustained Release of Saponins via Phytosomes

**Figure 1:**
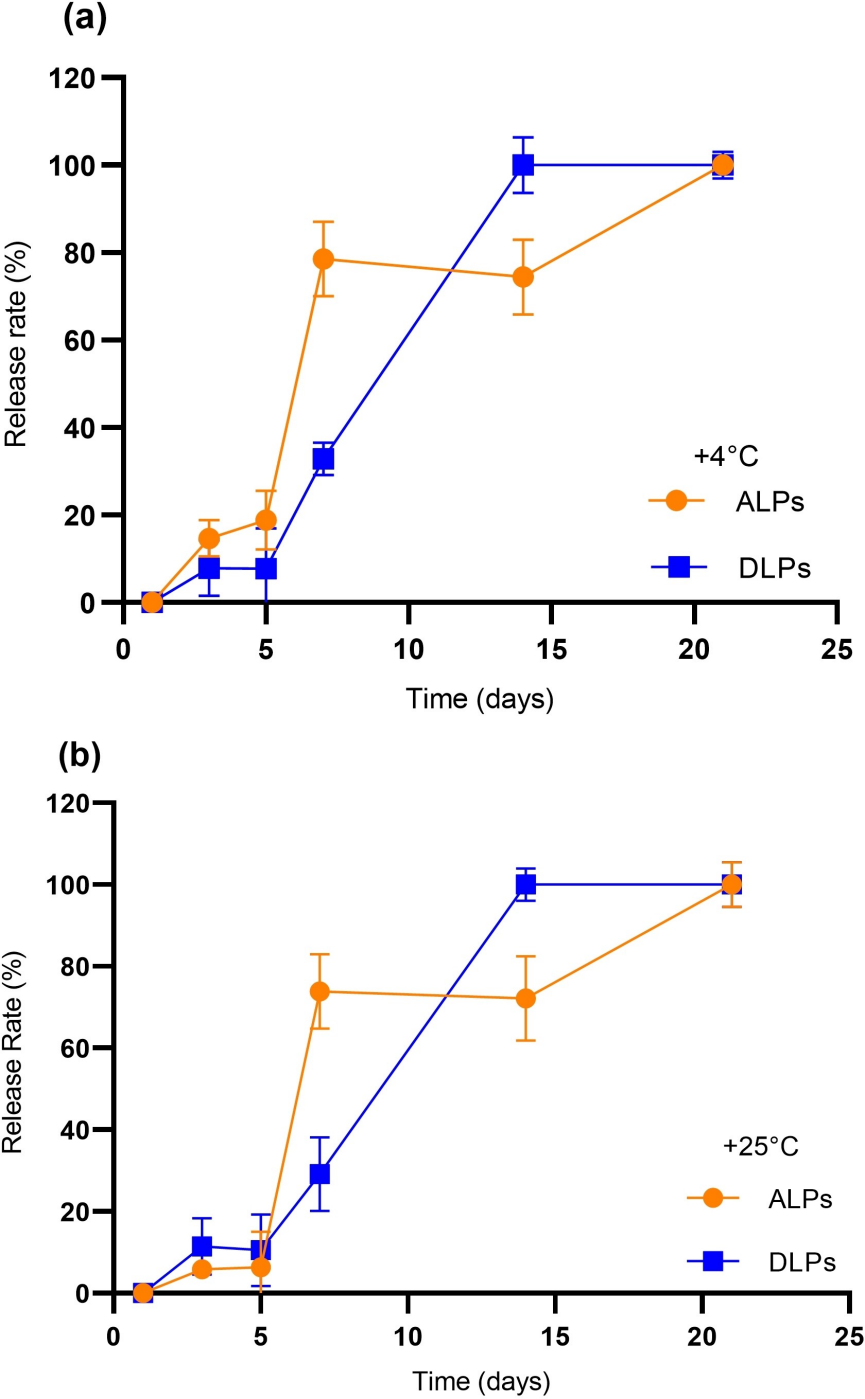
Release profiles of saponin‐loaded phytosomes. (a) Percentage release rate of saponins from ALPs and DLPs at +4 °C. (b) Percentage release rate of saponins from ALPs and DLPs at +25 °C.

Release studies of aristatoside‐C and davisianoside B from their phytosomal formulations were carried out at +4 °C and +25 °C. It was observed that phytosomal formulations demonstrated sustained release profiles for both saponins at +4 °C (Figure 1a) and +25 °C (Figure 1b). Saponin formulations presented similar release profiles at both temperatures. As all davisianoside B was released from DLPs on the 14th day of loading, aristatoside C was released from ALPs on the 21st day at both temperatures studied. As the aristatoside C release was faster on the 7th day of loading than davisianoside‐B, it presented a longer period of complete release, which is 21 days. Sustained release of saponins from phytosomal formulations indicates the release of a molecule at a predetermined and constant rate, enabling continuous drug delivery over a long period of time at a given concentration level. Our results indicate that phytosomal formulations of studied saponins may also be suitable for further sustained release of similar small drug molecules.^[26]^

### Stability Analysis of Phytosomal Formulations

**Figure 2:**
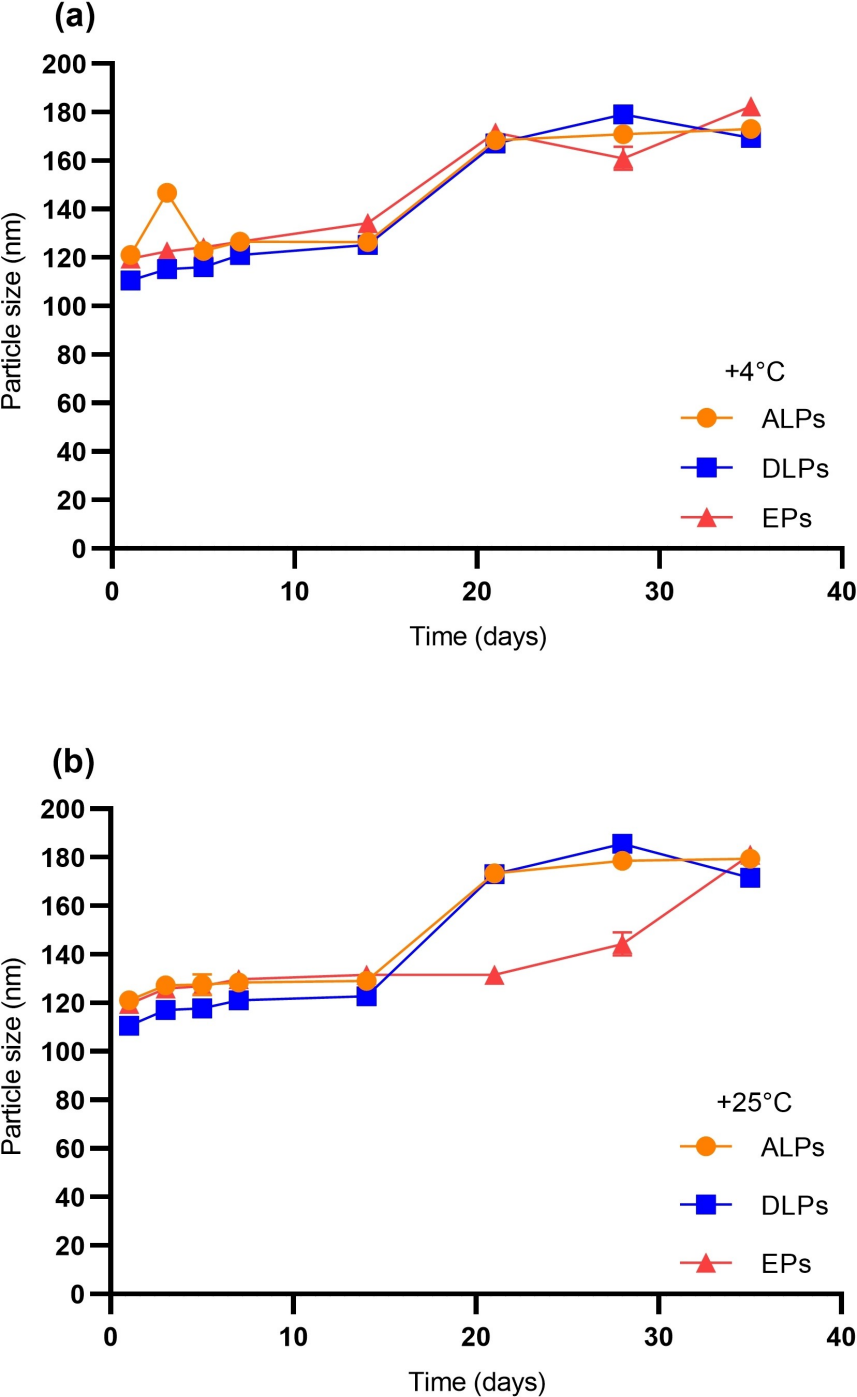
Time and temperature‐dependent stability analysis of saponin‐loaded phytosomes based on particle size characteristics. ALPs (orange line), DLPs (blue line), and EPs (red line). (a) Changes in the particle size of phytosomes stored at +4 °C. (b) Changes in the particle size of phytosomes stored at +25 °C.

**Figure 3:**
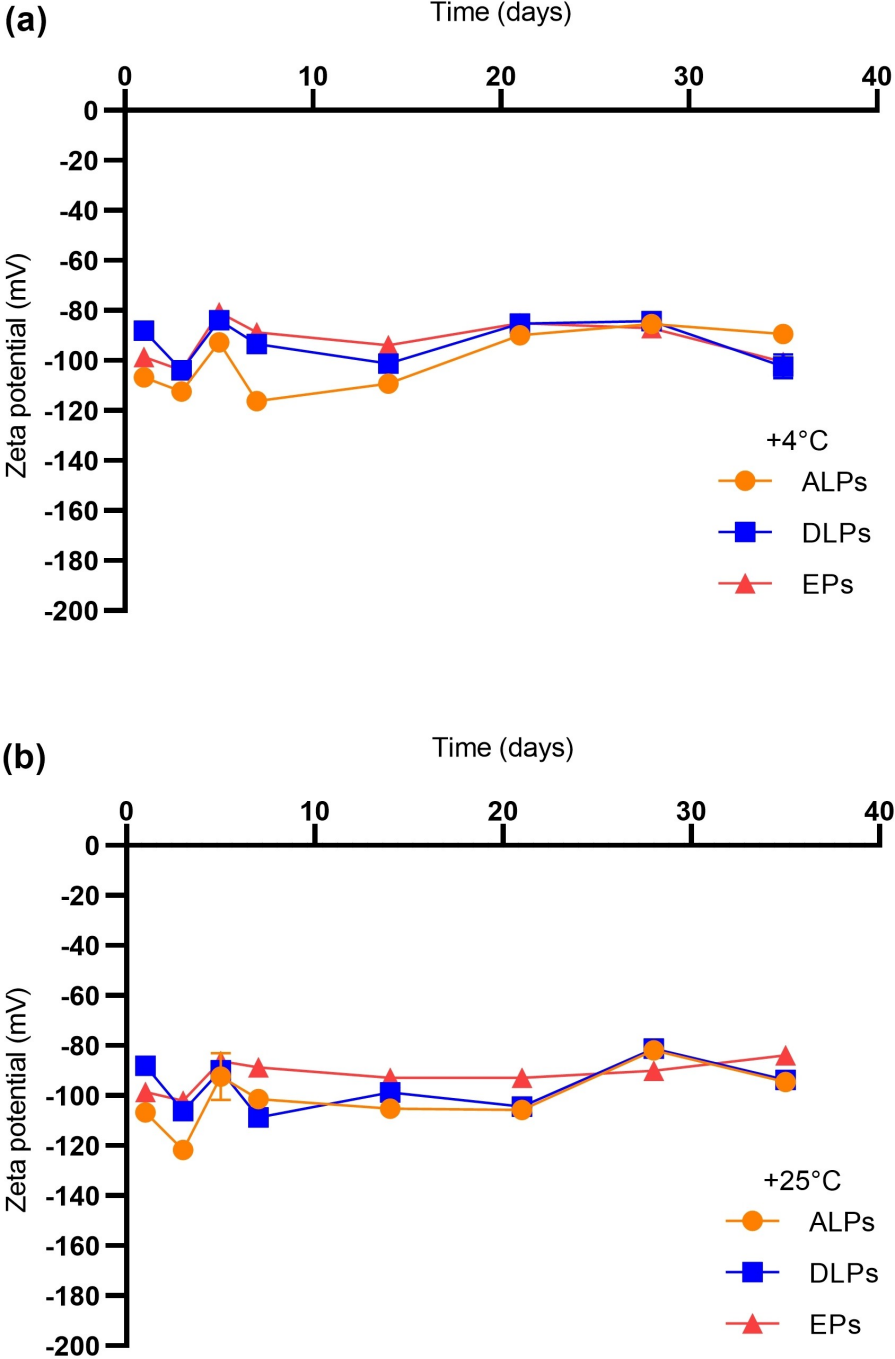
Time and temperature dependent stability analysis of saponin loaded phytosomes based on particle zeta potential. ALPs (orange line), DLPs (blue line), and EPs (red line). (a) Zeta potential of phytosomes stored at +4 °C. (b) Zeta potential of phytosomes stored at +25 °C.

Time and temperature‐dependent stability analysis of ALPs and DLPs were investigated by monitoring the hydrodynamic size changes and zeta potential of the nanovesicles. The particle size of formulations stored at +4 °C (Figure 2a) and +25 °C (Figure 2b) remained stable over 14 days. The hydrodynamic size of the phytosomal formulations increased after 14 days, indicating that nanovesicles have lost their stability after two weeks at studied temperatures. Particle size measurements of ALPs and DLPs showed that the particles had a low polydispersity index (data not shown) and had remained monodisperse throughout the experiment. It was shown that saponin‐loaded phytosomal formulations do not vary in size, which is important for successful delivery of therapeutic molecules. The zeta potential of ALPs and DLPs stored at +4 °C (Figure 3a) and +25 °C (Figure 3b) were measured at different time intervals for up to 35 days. As with the particle size data, zeta potential values did not change significantly at either temperature. This can be correlated with the hydrodynamic size differences of particles within 35 days period. Since there were no excessive changes in particle size values, the zeta potentials of phytosomes remained stable as well.

### In Vitro Hemolytic Activity

**Figure 4:**
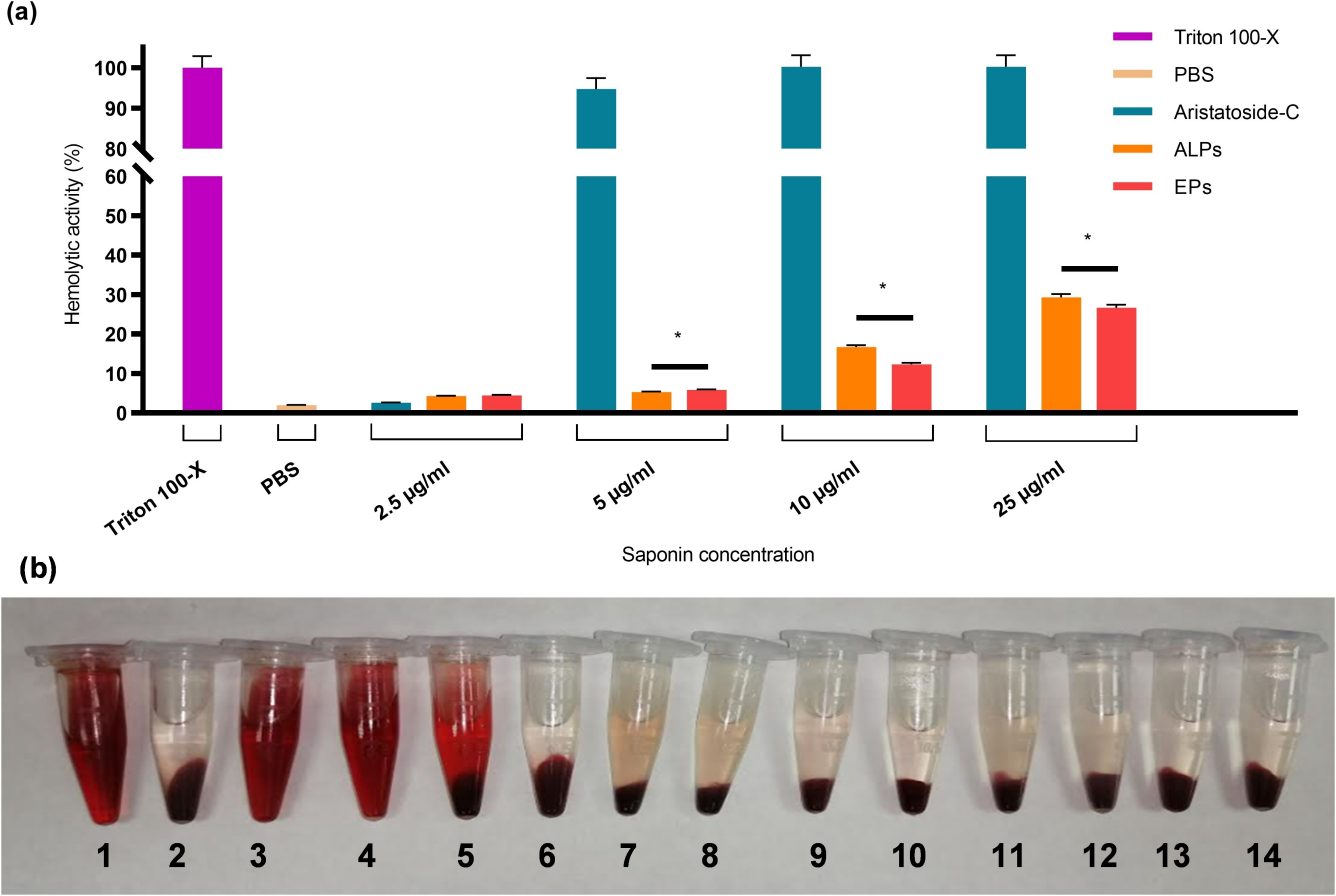
Hemolytic activity analysis of free/encapsulated aristatoside C saponin and empty phytosomes. (a) The percentage of hemolytic activity of the ALPs was analyzed with the spectrophotometric method (Hemoglobin absorbance at 540 nm). Triton X‐100 is the positive control, and PBS is the negative control. (b) Qualitative hemolytic activity analysis of ALPs. **1**. Triton X‐100. **2**. PBS. Free aristatoside C concentrations, **3**. 25 μg/ml, **4**. 10 μg/ml, **5**. 5 μg/ml, **6**. 2,5 μg/ml, aristatoside C concentrations of ALPs **7**. 25 μg/ml, **8**. 10 μg/ml, **9**. 5 μg/ml, **10**. 2,5 μg/ml. Empty phytosomes (EPs) were diluted to be used in corresponding concentration of saponin‐loaded phytosomes. **11**. 25 μg/ml, **12**. 10 μg/ml, **13**. 5 μg/ml, **14**. 2,5 μg/ml. ALPs: Aristatoside C loaded phytosomes; EPs: Empty phytosomes. * p<0,05.

**Figure 5:**
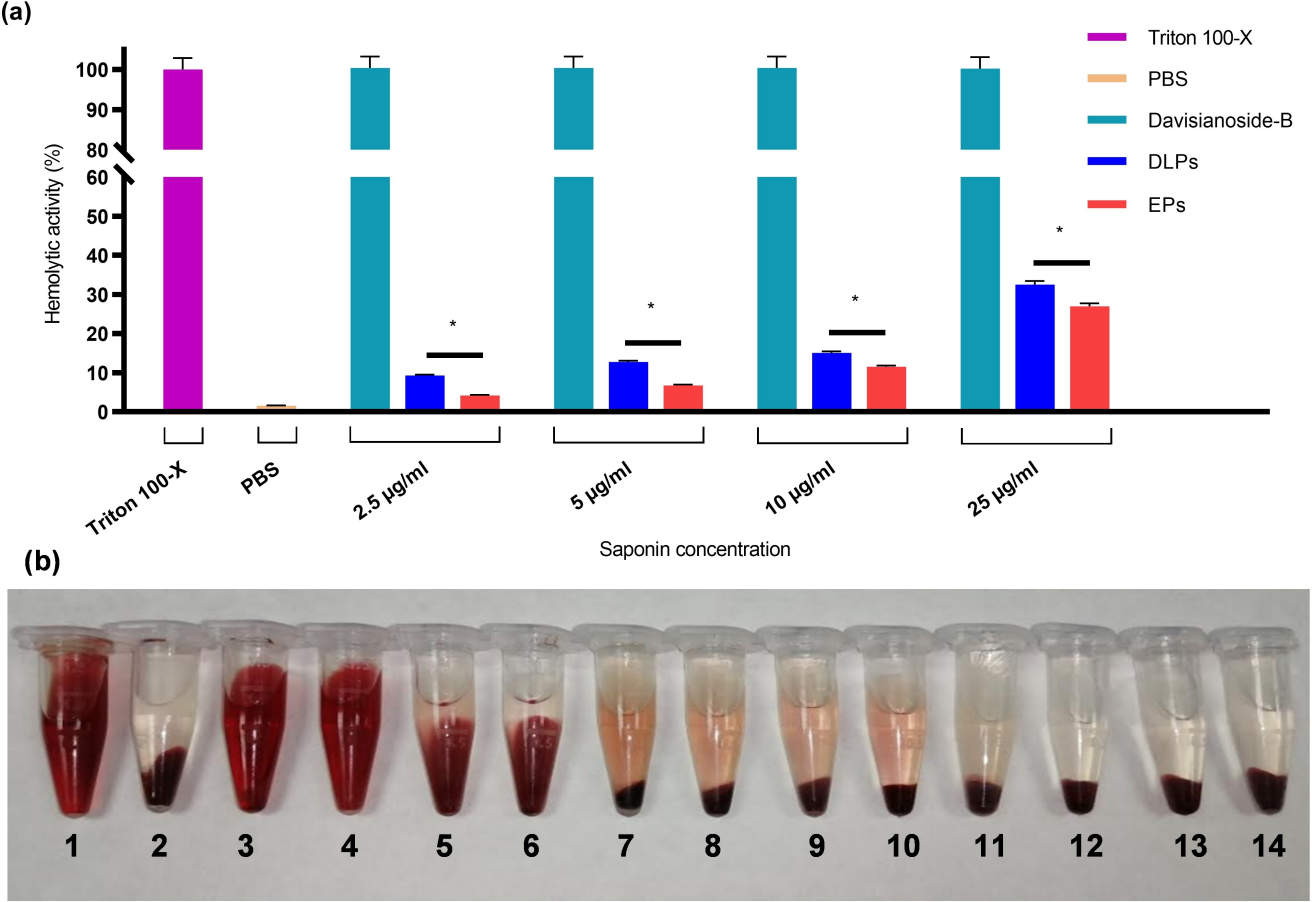
Hemolytic activity analysis of free/encapsulated davisianoside B saponin and empty phytosomes. (A) The percentage of hemolytic activity of the DLPs was analyzed with the spectrophotometric method (Hemoglobin absorbance at 540 nm). Triton X‐100 is the positive control, and PBS is the negative control. (B) Qualitative hemolytic activity analysis of DLPs. **1**. Triton X‐100. **2**. PBS. Free davisianoside B concentrations **3**. 25 μg/ml, **4**. 10 μg/ml, **5**. 5 μg/ml, **6**. 2,5 μg/ml, davisianoside B concentrations of DLPs **7**. 25 μg/ml, **8**. 10 μg/ml, **9**. 5 μg/ml, **10**. 2,5 μg/ml. Empty phytosomes (EPs) were diluted to be used in corresponding concentration of saponin‐loaded phytosomes.**11**. 25 μg/ml, **12**. 10 μg/ml, **13**. 5 μg/ml, **14**. 2,5 μg/ml. DLPs: Davisianoside B loaded phytosomes; EPs: Empty phytosomes. * p<0,05.

Saponins are molecules well‐known for their hemolytic activity, which makes them difficult to use in clinic. In this context, to reduce the hemolytic activity of aristatoside C and davicyanoside‐B, they were loaded into phytosomes. The qualitative and quantitative hemolytic activity of ALPs, DLPs, and EPs was assessed using corresponding free saponins as controls. ALPs and DLPs were diluted to obtain gradient saponin concentrations, while EPs were diluted according to the corresponding saponin‐loaded phytosomes. The hemolytic activity of the ALPs, DLPs, and EPs was determined by quantitatively by spectrophotometric evaluation of haemoglobin release from erythrocytes treated with the phytosomal formulation (Figure 4 and Figure 5). The percentage of hemolytic activity value obtained by the quantitative analysis for 2,5, 5, 10, and 25 μg/ml saponins were analyzed. It was also assessed qualitatively by observing the degradation of blood cells (Figure 4b and Figure 5b). Hemolytic activity of saponin‐loaded phytosomes was measured as 1 % for ALPs and 10 % for DLPs at 2,5 μg/ml concentration of corresponding saponin (Figure 4a and Figure 5a). The results of the qualitative and quantitative analysis of the hemolytic activity of ALPs and DLPs showed that the saponins had almost no hemolytic activity compared to their free form, thereby making them suitable for intravenous administration.

### Cytotoxicity Assay

**Figure 6:**
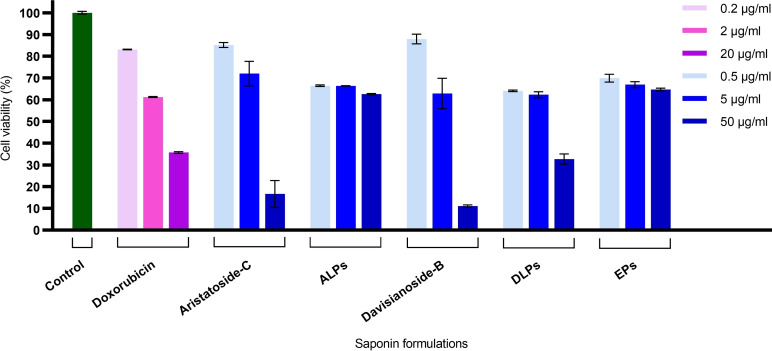
The cell viability of the A‐549 cell line by the MTT assay. Cells were incubated with ALPs and DLPs for 48 hours. The X‐axis shows the concentrations of free and encapsulated saponin inside the phytosomal formulations. EPs were diluted to employ the same amount of empty phytosome, proportional to the concentration of saponin‐loaded phytosomes. ALPs, aristatoside C loaded phytosomes. DLPs, davisianoside B loaded phytosomes. EPs, empty phytosome.

Previously, it was reported that the cytotoxicity studies of aristatoside C and davisianoside B presented higher cytotoxic activity than doxorubicin against lung cancer cell line A‐549 using the MTT method.^[27]^ Our results showed that free aristatoside C and davisianoside B exhibited a similar decrease in cell viability as doxorubicin at the concentrations studied (Figure 6). Here, we obtained similar cytotoxic activity for free aristatoside C and davisianoside B with IC_50_ values of 11,43±3,36 μg/ml and 7,71±1,65 μg/ml, respectively (Table 3). Encapsulation of saponins to phytosomes resulted similar cytotoxic activity for DLP (IC_50_ 9,64±0,02 μg/ml) compared to free davisianoside B (IC_50_ 7,71±1,65 μg/ml). This is a promising result for the phytosomal formulation of davisianoside‐B, which does not interfere with its cytotoxic activity as it provides a dramatic reduction in its hemolytic activity. There is a rather low cytotoxic activity of ALPs (IC50<50 μg/ml) in comparison to free aristatoside‐C. EPs also showed low cytotoxic activity against A‐549 cells. The reason why ALPs do not show cytotoxic effects would be the following.

**Table 3:** IC_50_ values for ALPs and DLPs in A‐549 cell line.

Aristatoside‐C	Davisianoside‐B	ALP^[a]^	DLPs^[b]^	EPs^[c]^
11,43±3,36	7,71±1,65	<50	9,64±0,02	<50

[a] ALPs: Aristatoside C loaded phytosomes. [b] DLPs: Davisianoside B loaded phytosomes. [c] EPs: Empty phytosomes (μg/ml).

Their zeta potential should be either above +30,0 mV or below −30,0 mV, as is known for stable nanocarriers.^[25]^ Thus, phytosomes have stable properties based on the zeta potential of ALPs (−106,78±0,39 mV) and DLPs (−88,1±2,85 mV) at room temperature, where ALPs should be rigid in comparison to DLPs. This may be one of the reasons why the encapsulation efficiency of ALPs (68,0 %) is lower than that of DLPs (79,5 %). Both phytosomes are stable over 35 days at +4 °C and +25 °C with a slight change in their size and almost no change in their zeta potential. Saponin release profiles showed that a complete aristatoside C release was complete after 21 days, and davisianoside B was complete after 14 days. Saponins took longer to be released from ALPs than from DLPs. The fact that aristatoside C contains an additional sugar unit in its structure compared to davisianoside‐B, was thought to be the reason for the reduced cytotoxic activity of the phytosomal formulations. The primary cause of this reduction is likely to be the cytotoxic activity associated with the interactions between sugars and lecithin‐cholesterol molecules rather than the amount of sugar. Furthermore, the additional rhamnose sugar on the exterior of the davisianoside B structure may reduce the strength of this interaction. These results support the hypothesis that ALPs have a more rigid structure than DLPs, leading to a significant reduction in their relative cytotoxic activity.

## Conclusions

In the present study, phytosomal formulations of aristatoside C and davisianoside B were prepared using the thin film hydration method to enhance the hemocompatibility of two plant‐based saponins with sustained release profiles over 21 and 14 days, respectively. Analysis of DLS has showed that phytosomal saponin formulations have a hydrodynamic size of 110 nm (DLPs) and 120 nm (ALPs), which are suitable sizes for biomedical applications. Polydispersity indexes under 0,5 indicate monodispersed nanovesicles. Thus, the obtained PDI values around 0,250 from DLS confirm the phytosomal saponin formulations are highly monodispersed in the mixture, leading to a fine size distribution of the phytosomal formulation. PALS analysis revealed that phytosomes have a high negative surface charge, which increases the stability and dispersity of nanovesicles and prevents them from aggregation. Stability analysis of phytosomal saponin formulations has demonstrated that phytosomes are stable for 35 days. Release kinetic studies of saponins from phytosomes showed that ALPs and DLPs have sustained release profiles over 21 and 14 days, respectively. Combined with the stability results of both saponin formulations, it raised a great prospect for their use as a delivery system for prolonged or sustained‐release vehicles.

Based on both qualitative and quantitative analysis of the hemolytic activity of free and phytosomal formulations of saponins, it was presented that encapsulation of aristatoside C and davisianoside B to phytosomes reduces their hemolytic activity dramatically. Qualitative analysis of hemocompatibility has shown that free saponins cause hemolysis, while this effect was eradicated through their formulations into phytosomes. Quantitative analysis of hemolytic activity of both ALPs and DLPs confirms these results. Encapsulation of saponins to phytosomes presented similar cytotoxic activity for DLP compared to free davisianoside‐B, as aristatoside C did not show cytotoxic activity in its phytosomal formulation (ALPs). This is a promising result for phytosomal formulation of davisianoside B that does not change its cytotoxic activity as it dramatically decreases its hemolytic activity.

In conclusion, the encapsulation of aristatoside C and davisianoside B to phytosomes has been achieved for the first time. Phytosomal formulations of aristatoside C and davisianoside B with small size, uniform distribution and high stability were prepared successfully. *In vitro* release study showed a slow and sustained release of both saponins from phytosomal formulations. They presented almost no hemolytic activity while retaining the cytotoxic activity of DLPs as in their free form. In conclusion, we have successfully developed a vesicular delivery vehicle for davisianoside B as a promising nanotherapeutic with reduced hemolytic activity and a sustained release profile, which will be a guide for researchers studying its anti‐cancer effects in detail for clinical translation.

## Experimental Section

### Material

Soybean lecithin (≥99,9 %) and cholesterol (≥99,0 %) were purchased from Sigma Aldrich (St Louis, MO, USA). Absolute methanol (≥99,8 %) and absolute chloroform (≥99,8 %) were purchased from Merck (Massachusetts, MA, USA). Amicon 10 kDa Ultra‐centrifugal filter was purchased from Millipore (Burlington, MA, USA). Triton X‐100 was purchased from Sigma Aldrich (St Louis, MO, USA). Phosphate buffer saline (PBS) (0,1 M pH:7,2). A‐549 (human alveolar adenocarcinoma) cell line used to test cytotoxic activity. The A‐549 cell line was purchased from the American Type Culture Collection (ATCC, Manassas, VA). Cells were cultivated in Dulbecco's Modified Eagle Medium (DMEM/F‐12) supplemented with 10 % Fetal Bovine Serum (FBS) and 0,1 % penicillin‐streptomycin (Pen‐Strep) solution. Cells were incubated at 37 °C in a 95 % humidified atmosphere with 5 % CO_2_.

### Isolation of Aristatoside C and Davisianoside‐B

**Figure 7:**
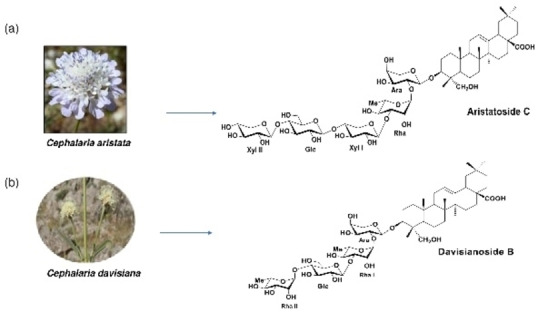
The structures of isolated saponins from (a) *Cephalaria aristate* and (b) *Cephalaria davisiana*.

The aristatoside C (≥99,0 %) and davisianoside B (≥99,0 %) saponins were previously isolated, purified, and structurally determined[^13^, ^28^, ^29^] from the plant *Cephalaria aristata* C. *Koch* and *Cephalaria davisiana Gokturk & Sumbul* respectively. These are structurally new saponins, isolated from *Cephalaria* species that have been gained to the literature[^28^, ^29^] (Figure 7). Both are monodesmosidic saponins and amorphous compounds. Their aglycone type is hederagenin.

### Preparation and Characterization of Phytosomes

#### Preparation of Aristatoside C and Davisianoside B Phytosomes

**Figure 8:**
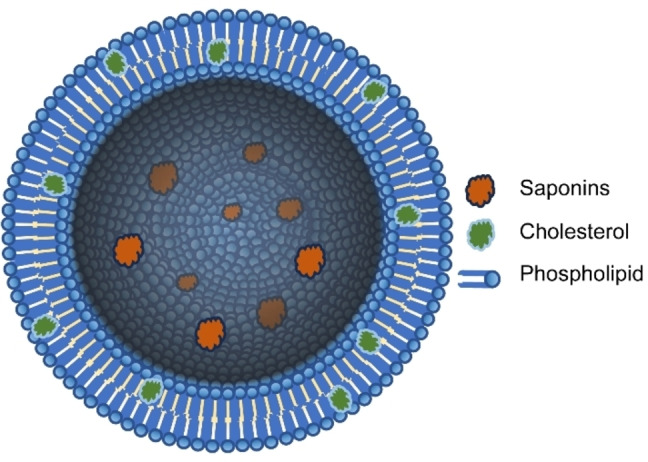
Schematic illustration of saponin‐loaded phytosome.

The ALPs and DLPs were synthesized by the thin film hydration technique, as described by Nagpal et al.^[30]^ A mixture of soy lecithin and cholesterol (1,8 : 1,0 mM) was prepared and dissolved in a solvent mixture of chloroform and methanol (2 : 1) (Table 4). The solution was then placed in a round‐bottomed rotary flask and subjected to evaporation in a rotary evaporator (Heidolph, Germany) at 90 rpm and 37 °C until a thin film was formed. The thin film was hydrated using a mixture of aristatoside C and davisianoside B (0,5 mg/ml), each dissolved separately in 4 ml of double‐distilled water (Table 4). The hydration process was carried out at a speed of 270 rpm and a temperature of 45 °C for 2 hours (Figure 8). Once the thin film was completely dissolved, the mixture was sonicated using an ultrasonic bath (ISOLAB, Wertheim, Germany) for 10 minutes. The mixture was then sonicated in a probe sonicator for 11 minutes, divided into two sets of 6 and 5 minutes respectively, with a 10 % amplitude, 5‐second pulse on, and 5‐second pulse off pattern (Branson, USA). The resulting ALPs and DLPs were stored in the dark conditions at +4 °C.

**Table 4:** Ingredients and amounts in ALPs and DLPs formulations.

Component	ALPs^[a]^	DLPs^[b]^
Soybean lecithin (mM)	17,47	17,47
Cholesterol (mM)	9,7	9,7
Aristatoside C (mg)	2,0	–
Davisioniside‐B (mg)	–	2,0

[a] ALPs: Aristatoside C loaded phytosomes. [b] DLPs: Davisianoside B loaded phytosomes.

#### Particle Size and Zeta Potential Analysis

The vesicle size of the ALPs and DLPs was measured using the dynamic light scattering technique. The zeta potentials of the ALPs and DLPs were determined using phase analysis light scattering technique, as described by Xu et al..^[31]^ To measure the particle size, PDI, and zeta potential of the ALPs and DLPs, 100 μl of each sample was dispersed in 2 ml of double‐filtered deionized water. The measurements were performed using a NanoPartica SZ‐100 instrument (Horiba Scientific Ins., USA). Measurements were performed at a temperature of +25 °C and were performed in triplicate, according to the method described by Gokce et al..^[32]^

#### Encapsulation Efficiency (%EE)

The encapsulation efficiency (%EE) of ALPs and DLPs was determined spectrophotometrically. The %EE of the aristatoside C and davisianoside B in the phytosomes was assessed by separating the fraction of the non‐encapsulated molecules.^[33]^ 400 μL of ALPs and DLPs were placed in Amicon centrifugal type filters with a pore diameter of 10 kDa, Millipore (Burlington, MA, USA). They were then centrifuged at 14.000 rpm for 30 min (Allegra X‐30 Bechman Coulter; Brea, CA, USA). The resulting filtrate was separated, and the absorbance value was determined in the UV‐Vis spectrophotometer (Biochrom, Libra S22, UK).

(1) $$\%\mathrm{EE}=\frac{\begin{array}{c} Total\;amount\;of\;saponin-\\ Amount\;of\;free\;saponin \end{array}}{\mathrm{Total}\;\mathrm{amount}\;\mathrm{of}\;\mathrm{saponin}}\;\times \;100\;$$

Aristatoside C was measured at its maximum absorption point of 214 nm, while davisianoside B was measured at 215 nm. The percentages of the encapsulation efficiency of the ALPs and DLPs were calculated according to *(eq. 1)*.

#### In vitro Release Assay of Saponins from ALPs and DLPs

A modified protocol from the literature^[34]^ was followed to study the rate of release of saponins from the phytosomes. In this method, 1 ml of ALPs and DLPs was introduced into a dialysis bag, that was tightly sealed at both ends. The bag was placed in a beaker containing 30 % (v/v) ethanol in 50 ml of PBS with a pH of 7,4. The beaker was maintained at a temperature of 37 °C using a magnetic stirrer (Heidolph, MR3002G, Germany) operating at a constant speed of 150 rpm. Samples were incubated under these conditions for 48 hours. At specified time intervals, 1 ml of the drug released into the PBS medium was withdrawn, and an equal volume of fresh PBS buffer was immediately added to maintain sink conditions. The withdrawn samples were analyzed using a UV‐visible spectrophotometer (Biochrom, Libra S22, UK) at 214 nm for aristatoside C and 215 nm for davisianoside‐B. The percentage of released aristatoside C and davisianoside B in the PBS buffer was calculated as the cumulative percentage release. Experiments were performed in triplicate to ensure the reproducibility of results and data were expressed as average±standard deviation (SD).

#### Stability of ALPs and DLPs

The stability of the ALPs and DLPs was assessed at +4 °C and +25 °C, over 35 days. During this period, the physical changes of the ALPs and DLPs, including particle size, zeta potential, and encapsulation efficiency were investigated. The study conducted by Sahin et al.^[35]^ provided insight into these parameters.

#### Preparation of Erythrocyte Suspension

Four milliliters of whole blood were obtained from healthy volunteers and transferred into tubes containing EDTA buffer. The experimental protocol was approved by the Human Ethics Committee of Ege University, and all procedures were performed according to the principles outlined in the Declaration of Helsinki. Before participation, the subjects were fully informed about the procedures and provided their informed consent by signing the appropriate consent forms (approval number: E.215344). The blood samples were then centrifuged at 3.000 rpm for 5 minutes at a temperature of 4 °C. This step was carried out to separate the plasma from the erythrocytes. The plasma was discarded and the resulting pellet containing the erythrocytes was washed twice with physiological saline solution at a pH of 7,2±0,2. After each wash, the samples were centrifuged again at 3.000 rpm for 5 minutes at 4 °C according to the method described by Arslan and Çelik.^[36]^

#### In vitro Hemolytic Activity

The *in vitro* hemolytic activity of phytosomes loaded with aristatoside C and davisianoside B was determined by the spectrophotometric method.^[8]^ The erythrocytes were diluted with 4 ml PBS and incubated with phytosomal formulations on a shaker at 37 °C for 1,5 hours. Both the ALPs and DLPs consist of the same amount of saponin (0,5 mg/ml). These phytosomes were diluted at different ratios with PBS to obtain gradients of saponin concentration in the samples. Phytosome formulations were diluted to obtain saponin concentrations of 2,5, 5, 10, 25 μg/ml. EPs were diluted equally for comparison with saponin‐loaded phytosomes. PBS and Triton X‐100 were used as controls. The sample was centrifuged at 3.000 rpm for 5 min at 4 °C. The amount of free hemoglobin in the supernatant was measured using a UV‐Vis spectrophotometer at 50 nm. Each experiment was performed in triplicate to ensure the reproducibility of the results. The degree of hemolysis percentage by the extracts was calculated according to the following formula (*eq.2*):

(2) $$\%\mathrm{Hemolysis}=\frac{A_{c}-\mathrm{At}}{A_{c}}\;\times \;100$$

Where A_t_ is the absorbance of ALPs and DLPs and A_c_ is the absorbance of Triton X‐100.

#### Cytotoxicity of ALPs and DLPs

The cytotoxic activity of the compounds was determined by the MTT [3‐(4,5‐dimethyl‐2‐thiazolyl)‐2,5‐diphenyl‐2H‐tetrazolium‐bromide)] assay.^[37]^ This colorimetric assay is widely used to determine cell viability and is based on the activity of mitochondrial reductases. A‐549 cells were cultivated in 96 well plates for 24 h at an initial concentration of 1×10^5^ at 37 °C in a humidified atmosphere with 5 % CO_2_. At the end of 24 h, the cells were treated with various doses of compounds (0,5, 5, 50 μg/ml) and incubated for 48 h at 37°C. Each well was then supplemented with 20 μl of MTT solution followed by incubation at 37 °C for 4 hours. The medium‐containing solution was removed from the wells and 150 μl of dimethyl sulfoxide (DMSO) was added to the wells to dissolve the formazan crystals. The optical density was measured at 570 nm using a UV‐Vis spectrophotometer (Thermo Multiskan Sky). The percentage of cell viability was determined using the following formula (*eq.3*):

(3) $$\%\;\mathrm{Viability}=\frac{At\;-\;Ab}{Ac\;-\;Ab}\;\times \;100$$

Where A_t_, is the absorbance of formazan on treated cells; A_c_, is the absorbance of formazan on control cells and A_b_, is the absorbance of blank.

#### Statistical Analysis

Experimental data are expressed as mean±standard error, the statistical differences of these data were determined by t‐test, ANOVA and post‐hoc tests were performed using SPSS and GraphPad Prism 8 software. Statistical significance in the analyses was determined by considering p‐values less than 0,05.

## Conflict of Interests

The authors declare no conflict of interest.

1

## Data Availability

The data that support the findings of this study are available from the corresponding author upon reasonable request.
